# Effect of the mixture of oleaster (*E. angustifolia* L.) and black cumin (*Nigella sativa*) flours as functional compounds on the quality characteristics of toast bread

**DOI:** 10.1002/fsn3.3430

**Published:** 2023-05-15

**Authors:** Saba Ghadarloo, Samar Mansouripour, Solmaz Saremnezhad

**Affiliations:** ^1^ Department of Food Science and Technology, Faculty of Pharmacy, Tehran Medical Sciences Islamic Azad University Tehran Iran

**Keywords:** antioxidant activity, black cumin, calcium, functional toast bread, oleaster, phenolic compounds

## Abstract

The present study was conducted to evaluate the addition of a mixture of oleaster (OL; *E. angustifolia* L.) and black cumin (BC; *Nigella sativa*) flours on the quality characteristics of toast bread. The concentration of OL and BC mixture (1:1 w/w ratio) was 0 (T1), 1.5% (T2), 2% (T3), and 2.5% (T4) of total flour content. The bread samples containing the mixture of OL and BC flours had more protein content (8.49%–9.65%) than the control (6.81%; *p* < .05). The highest phenolic compounds and DPPH free radical scavenging capacity were observed in T4 and T3 samples, respectively. The OL and BC flours decreased the brightness, yellowness, and chroma and increased the redness compared to the control. The mixed flour concentration influenced the bread's hardness and chewiness. Adding OL and BC flours increased the calcium content in the bread (467.65–600.41 ppm) compared to the control (363.9 ppm; *p* < .05). The OL and BC flour mixture created a more compact texture in the bread samples. In the sensory evaluation, there was not a significant difference between the overall acceptability of the bread containing the mixture of OL and BC flours and the control (*p* > .05). Finally, it is recommended to utilize a mixture of 2% of OL and BC flours in toast bread to improve its nutritional properties.

## Graphical abstract

The antioxidant activity of toast bread increased by the addition of the mixture of oleaster (OL) and black cumin (BC) flours. The bread samples containing the mixture of OL and BC had more protein and calcium content. The overall acceptability was satisfying and utilizing a mixture of 2% of OL and BC in toast bread is recommended concentration.

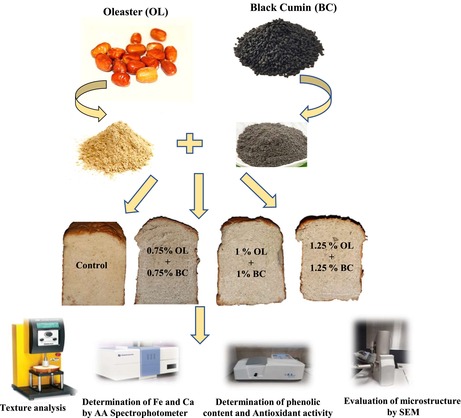

## 1 INTRODUCTION

Bread is one of the most widely consumed and staple foods in many countries worldwide (Đurović et al., 2020; Mikulec et al., 2020). Nowadays, consumers believe in the direct role of food in their health (Sahan et al., 2019) and tend to choose products with health‐enhancing characteristics (Mikulec et al., 2019). Hence, various formulations are being developed to answer the consumer's demands. In the past few years, some studies have been conducted to improve wheat bread's nutritional characteristics, using different substances with natural origins, such as fruits, vegetables, plant extracts, and seeds (Alkandari et al., 2019; Bourekoua et al., 2018; Mikulec et al., 2019, 2020; Özcan, 2022; Udomkun et al., 2022; Wandersleben et al., 2018).

Oleaster (*Elaeagnus angustifolia* L.; OL) is a tree that belongs to the Elaeagnaceae family and grows in a wide geographical area, including Asia and Europe. Oleaster is a valuable fruit due to the presence of phenolic and antioxidant compounds (Farzaei et al., 2015; Öztürk et al., 2018). Its fruit and seeds are used in traditional medicine to treat urinary diseases, diarrhea, nausea, asthma, arthritis, fever, pain, and kidney disorders (Sarraf et al., 2017).

Black cumin (*Nigella sativa*; BC) is a herbaceous plant from the Ranunculaceae family. This plant is found in Europe, Asia, the Middle East, North Africa, and the Mediterranean region (Jan et al., 2019; Mohebbati & Abbasnezhad, 2020). It is rich in constituents with antioxidant properties and contains protein, fat, carbohydrates, vitamins, and minerals (Amin & Hosseinzadeh, 2016; Hannan et al., 2021; Jan et al., 2019; Osman et al., 2015; Periasamy et al., 2016). BC is widely used in traditional medicine to treat various chronic diseases such as diabetes, high blood pressure, asthma, cancer, and cardiovascular diseases (Hannan et al., 2021; Periasamy et al., 2016).

Oleaster as a functional ingredient has been used in some food formulations. Scientific reports show the antioxidant activity in oleaster‐containing formulations. Adding oleaster flour to yogurt (Öztürk et al., 2018), ice cream (Çakmakçı et al., 2015), and cookies (Sahan et al., 2019) increased the total phenol content and antioxidant capacity. Nezamdoost‐sani et al. (2018) demonstrated that oleaster flour increased the total sugar and ash, and decreased the protein content in lavash bread (Iranian flatbread). Osman et al. (2015) added BC flour to flatbread and observed an increase in protein, fat, ash, and fiber contents. According to Al‐Ansi et al. (2019), the total phenol content, antioxidant activity, protein, fat, and ash increased in biscuits containing BC flour. To the best of our knowledge, the mixture of oleaster and BC flours has not been used in toast bread formulation. The present study aims to evaluate the effect of the simultaneous addition of oleaster and BC flours on the quality characteristics and nutritional properties of toast bread.

## 2 MATERIALS AND METHODS

### 2.1 Materials

The ingredients used to prepare the bread samples included wheat flour (82% extraction rate), sugar, milk, baker's yeast, improving agent (DATEM), salt, vegetable oil, oleaster, and BC. The improving agent was purchased from Sahar Co. and other ingredients were purchased from a local store. Oleaster (fruit and crust) and BC were ground and passed through a 60‐mesh sieve after removing the impurities and separating the seed from the oleaster.

All the chemicals purchased were of analytical grade (Merck). DPPH was obtained from Sigma‐Aldrich.

### 2.2 Preparation of toast bread

To produce the bread samples, wheat flour and powdered ingredients were first mixed with a mixer according to Table 1. Then, vegetable oil, milk, and a mixture of OL and BC flours were added. The mixing ratio of the OL and BC flours was as follows:

T1: Control sample (without OL and BC flours),
T2: sample with 1.5% mixture of OL and BC (0.75%: 0.75% w/w),
T3: sample with 2% mixture of OL and BC (1%: 1% w/w),
T4: sample with 2.5% mixture of OL and BC (1.25%: 1.25% w/w).

**TABLE 1:** Toast bread formulation.

Ingredient	Percentage (%flour weight)
Wheat flour	100
Sugar	10
Improving agent (DATEM)	1.12
Bakery yeast	4.8
Salt	2
Vegetable oil	1.99
Milk	64
The mixture of oleaster (OL) and black cumin (BC) flours	1.5–2.5

After mixing the ingredients, the dough mass was rested for 10 min in a proofer (Morshed Gohar) at 35°C and a relative humidity of 90%. Then, the dough was divided into pieces of about 650 g, poured into one‐third of the toast molds, and placed in the fermentation chamber for 30 min for final fermentation. Baking was performed in a rotary oven (Morshed Gohar) at 150–160°C for about 40 min. After cooling, the bread samples were sliced and packed in polyethylene bags (Hadidi et al., 2021).

### 2.3 Analysis of samples

Soxhlet (AOAC 935.38) and Kjeldahl (AOAC 950.36) methods were used for fat and protein measurement, respectively (AOAC, 2006). The moisture (AOAC 925.10) and ash content (AOAC 923.03) were determined by the gravimetric method (AOAC, 2006). Iron and calcium were measured according to Carocho et al. (2020) by atomic absorption spectrophotometry (AAS8020‐YOUNG LIN). All analyses were carried out for flours (wheat, oleaster, and BC) and bread samples in triplicate. The pH of bread was measured with a sevenCOMPACT—METTLER Swiss pH meter according to AACC 02‐52.01, and the specific volume was evaluated by the AACC‐approved method 10‐05.01 (AACC, 2000) in triplicate.

### 2.4 Determination of the total phenolic content (TPC)

Dried bread sample (3 g) was mixed with 30 mL of 80% ethanol on a magnetic stirrer (SCI FINETECH) for 30 min. Then, the contents were centrifuged at 1792 × *g* for 15 min (Pars Azma). The supernatant (20 mL) was mixed with 5 mL of hexane, stirred for 15 min, and then centrifuged for 20 min. The obtained supernatant, which contained hexane and fat, was gently separated by a syringe. The remaining solution was used to measure the concentration of phenolic compounds and evaluate the antioxidant activity (for each sample with three replicates). The total phenol content was determined according to the Folin–Ciocalteu method (Bourekoua et al., 2018). One milliliter of each extracted solution was mixed with 0.25 mL of Folin–Ciocalteu reagent and 10 mL of distilled water on a magnetic stirrer for 5 min. Then, 2 mL of sodium carbonate 7.5% (w/v) was added to the mixture and incubated in the dark for 60 min. The absorbance was measured using a Ultraviolet–Visible spectrophotometer (SU‐6100‐Philler Scientific) at 750 nm. The result was expressed as mg GAE/g d.m.

### 2.5 Antioxidant activity

The antioxidant activity of samples was determined using DPPH free radical scavenging method in triplicate (Piechowiak et al., 2020). The extracted solution (0.1 mL) containing phenolic compounds (from the total phenolic determination step) was mixed with 3.9 mL of 0.1 mM DPPH and incubated in the dark for 30 min at room temperature. Then, the absorbance was measured by a Ultraviolet–Visible spectrophotometer (SU‐6100‐Philler Scientific) at 510 nm.

### 2.6 Texture analysis

The texture profile analysis of bread crumb samples was determined using a texture analyzer (CT3 10K). The test was performed on the first, fourth, and sixth days after baking. The center of the bread was sliced into 2 × 2 × 2 cm cubes and compressed to 40% of the initial height at a testing speed of 1 mm/s with a 3.0 s of delay between the first and second compressions. Hardness, springiness, cohesiveness, and chewiness were measured on six repetitions (Bourekoua et al., 2018).

### 2.7 Color measurement

The changes in the color values *L* ^*^ (0 = black, 100 = white), *a* ^*^ (+: red; −: green), and b^*^ (+: yellow; −: blue) were determined in triplicate using Color Flex Hunter colorimeter in triplicate. The total color difference with control (Δ*E*) and chroma (color intensity) were calculated based on Equations 1 and 2, respectively (Çakmakçı et al., 2015; Mikulec et al., 2019).

(1) $$∆E=\sqrt{{\left(\mathrm{\Delta L}\right)}^{2}+{\left(\mathrm{\Delta a}\right)}^{2}+{\left(\mathrm{\Delta b}\right)}^{2}}$$

where Δ*L*, Δ*a*, and Δ*b* are the lightness, redness, and yellowness differences of samples, respectively

(2) $$C^{*}=\sqrt{a{*}^{2}+b{*}^{2}}$$

### 2.8 Microstructure

The microstructure was evaluated using scanning electron microscopy (SEM; MIRA3‐TESCAN). After drying with a freeze dryer (Christ alpha 1‐2 D plus), the bread sample was placed on a metal stand and covered with gold under vacuum by a desk sputter coater. The images were obtained at 5.00, 3.00, and 2.00 kx and a voltage of 15 kV (Espinosa‐Ramírez et al., 2020).

### 2.9 Sensory evaluation

Sensory evaluation was performed by 30 panelists (15 women and 15 men) using a nine‐point hedonic scale for taste, odor, after‐taste, color, texture, and overall acceptance. Bread samples with a three‐digit code were randomly provided to the panelists. The evaluation was done from strong dislike to extremely like, which were scored from 1 to 9, respectively (Mikulec et al., 2020).

### 2.10 Statistical analysis

The statistical analysis of the results was conducted using MINITAB16 software. The data were analyzed using a one‐way analysis of variance. Means were compared using Tukey's test with a significance level of *p* < .05.

## 3 RESULTS AND DISCUSSION

### 3.1 Flour characteristics

The physicochemical and antioxidant characteristics of the three flours used in this study are represented in Table 2. BC flour had significantly higher ash, calcium, iron, protein, and fat than the other two flour types. The results are in line with the values reported by Mamun and Absar (2018) and Jan et al. (2019). The highest phenolic content and antioxidant activity were observed in BC flour, followed by OL and wheat flour. According to Jan et al. (2019), the phenolic content and antioxidant activity of BC flour were 21.04 ± 0.72 mgGAE/g.d.m and 81.76 ± 1.51%, respectively, which was close to the present study. The lowest amount of protein was observed in OL flour (5.34%). Similar results have been reported by Sarraf et al. (2017; 6.64 ± 0.29) and Sharifian‐Nejad and Shekarchizadeh (2019; 5.79 ± 0.45).

**TABLE 2:** Physiochemical composition and antioxidant characteristics of oleaster flour (OL), black cumin flour (BC), and wheat flour (WF).

Flour	Moisture (%)	Ash (%)	Total phenolic content (mgGAE/g.d.m)	DPPH scavenging activity (%)	Protein (%)	Fe (mg/kg)	Ca (mg/kg)	Lipids (w/w%)
OL	7.46 ± 0.60^b^	1.53 ± 0.33^b^	16.44 ± 0.9^b^	69.35 ± 1.00^b^	5.35 ± 0.05^c^	13.64 ± 1.29^c^	526.8 ± 25.4^b^	0.47 ± 0.05^b^
BC	6.65 ± 0.27^b^	4.23 ± 0.71^a^	38.19 ± 1.34^a^	90.43 ± 0.18^a^	20.91 ± 0.11^a^	89.13 ± 2.28^a^	5149.90 ± 30.08^a^	38.74 ± 3.62^a^
WF	11.25 ± 0.31^a^	0.84 ± 0.11^b^	0.07 ± 0.01^c^	8.18 ± 0.57^c^	10.91 ± 0.32^b^	29.52 ± 1.75^b^	0.1 ± 0^c^	1.88 ± 0.04^b^

*Note*: Results are presented as a mean value ± SD, the same superscript letters are not significant in the same column (*p* > .05).

### 3.2 Physicochemical properties of breads

The physicochemical properties of bread samples are shown in Table 3. There was no significant difference in moisture content (*p* < .05). Despite the high amount of fiber in OL (Öztürk et al., 2018) and BC (Al‐Ansi et al., 2019), it seems that the insignificant change in moisture content of bread samples is related to the use of low concentration of these flours in the bread formulations. According to the reports of Sarraf et al. (2017), adding OL flour lower than 15% to donuts did not cause a significant difference in moisture content. An increase in the BC flour content in biscuit formulation elevated the moisture level of the product (Al‐Ansi et al., 2019).

**TABLE 3:** Physicochemical properties of bread samples.

Sample	Moisture (%)	Ash (%)	pH	Specific volume (cm^3^/g)	Protein (%)	Fe (ppm)	Ca (ppm)
T_1_	33.5 ± 1.65^a^	0.68 ± 0.02^b^	5.15 ± 0.02^b^	3.91 ± 0.92^a^	6.81 ± 0.3^c^	7.6 ± *0.02* ^a^	363.9 ± 7.6^c^
T_2_	35.6 ± 0.22^a^	0.78 ± 0.02^a^	5.22 ± 0.02^a^	3.86 ± 0.13^a^	8.49 ± 0.22^b^	7.65 ± 0.16^a^	467.65 ± 0.45^b^
T_3_	33.91 ± 0.66^a^	0.79 ± 0.01^a^	5.23 ± 0.01^a^	4.34 ± 0.34^a^	8.83 ± 0.13^b^	7.11 ± 0.13^b^	600.41 ± 1.51^a^
T_4_	34.26 ± 2.07^a^	0.77 ± 0.04^a^	5.21 ± 0.03^a^	4.04 ± 0.07^a^	9.65 ± 0.2^a^	6.96 ± 0.04^b^	577.2 ± 22.5^a^

*Note*: T_1_: Control, T_2_: 0.75% OL flour + 0.75% BC flour, T_3_: 1% OL flour + 1% BC flour, T_4_: 1.25% OL + 1.25% BC flour. Results are presented as a mean value ± SD, the same superscript letters are not significant in the same column (*p* > .05).

In the current study, OL and BC flours increased the ash content (Table 3; *p* < .05). Similar results have been reported for increasing the ash content of lavash bread (Nezamdoost‐sani et al., 2018) and ice cream (Çakmakçı et al., 2015) after using OL flour in the formulation. According to Al‐Ansi et al. (2019) and Osman et al. (2015), BC increased the ash content of biscuits and bread, respectively. BC flour contains various minerals, including iron, manganese, copper, magnesium, and zinc (Mamun & Absar, 2018). Oleaster also contains calcium, magnesium, potassium, iron, and manganese (Çakmakçı et al., 2015).

The pH increased significantly by adding OL and BC flours compared to the control (*p* < .05). The reason can be related to the antimicrobial characteristics of BC and oleaster flours (Farzaei et al., 2015; Osman et al., 2015) and their slightly negative effect on fermentation by *Saccharomyces cerevisiae*.

The results of the specific volume (3.86–4.34 cm^3^/g) in Table 3 demonstrate no significant difference (*p* > .05) among samples. Disulfide bonds from sulfur amino acids (methionine and cysteine) of flour improve the gluten quality and help to the better leavening of bread dough (Tao et al., 2018). The average content of sulfur amino acids in BC, OL, and wheat flour has been reported 0.5, 0.83, and 2.83 mg/g, respectively (Artikova et al., 2020; Kabir et al., 2019; Litwinek et al., 2013). In the present study, the results of specific volume indicate that probably the sulfur amino acids were not able to strengthen the gluten network enough to increase the specific volume significantly. According to the study conducted by Sarraf et al. (2017), there was no significant difference in the volume of donuts adding OL flour up to 3%, and utilizing more amounts of OL flour decreased the volume. There is also a report about a decrease in the specific volume of gluten‐free bread by partial substitution of rice flour with BC and sesame flours in higher amounts than concentrations used in the present study (Al‐Subhi, 2014). Hence, using low flour concentrations (below 3%) in the present study did not cause a significant difference in the specific volume of bread samples.

The OL and BC significantly increased the protein content of bread samples (*p* < .05). According to Nezamdoost‐sani et al. (2018), the protein decreased as the OL flour increased in lavash bread. In the present study, a higher amount of protein in BC flour compared to that of OL flour (Table 2) caused the increase of the protein content from 6.81% in the control to 9.65% in sample T4. Osman et al. (2015) also reported an increase in the protein content of flat bread by using BC flour in the dough.

An increase in the percentage of OL and BC flours caused a significant decrease in iron and a significant increase in calcium (*p* < .05; Table 3). The samples containing 1% and more OL and BC flours had lower iron content. It seems that it is due to the lower iron content of OL flour than wheat flour (Table 2). An increase in the calcium content of bread from 363.9 mg/kg in control to 467.65–600.41 mg/kg (in T2–T4) was observed with the increase in the concentrations of OL and BC flours. The amount of calcium in OL (526.8 mg/kg) and BC flours (5149.90 mg/kg) was much higher than the wheat flour (0.1 mg/kg; Table 2). BC flour contains high amounts of calcium (Mamun & Absar, 2018). The calcium in BC flour in the present study was nine times more than that of OL flour. Hence, an increase in the calcium content of toast bread was more related to the presence of BC flour.

### 3.3 Total phenolic compounds

**FIGURE 1:**
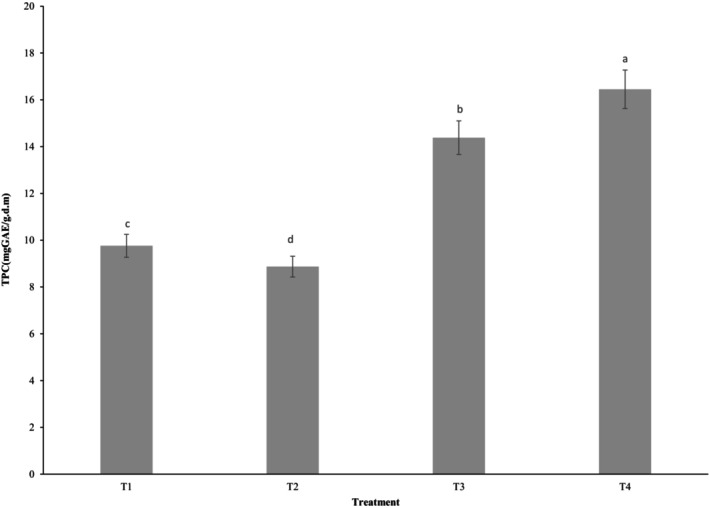
Total phenolic content of breads containing a mixture of oleaster and BC flours. T_1_: Control, T_2_: 0.75% OL flour + 0.75% BC flour, T_3_: 1% OL flour + 1% BC flour, T_4_: 1.25% OL + 1.25% BC flour. Results are presented as a mean value ± SD, the different superscript letters are significant (*p* < .05).

Figure 1 depicts the total phenolic content of bread samples. As the OL and BC content increased, the TPC in toast bread increased from 9.76 mgGAE/g.d.m (T1) to 16.45 mgGAE/g.d.m (T4). The oleaster contains phenolic compounds such as catechin, epicatechin, gallocatechin, quercetin, kaempferol, luteolin, isorhamnetin, terpenoids, phenolic acids (such as 4‐hydroxybenzoic acid, 4‐hydroxycinnamic acid, benzoic acid, caffeic acid, ferulic acid, and vanillic acid), chlorogenic acid, and gallic acid (Hamidpour et al., 2017; Hassanzadeh & Hassanpour, 2018; Öztürk et al., 2018; Sharifian‐Nejad & Shekarchizadeh, 2019). BC is also rich in compounds such as quercetin, kaempferol, isorhamnetin, pigments, resins, waxes, tannins, coumarins, thymoquinone, thymohydroquinone, dithymoquinone, thymol and carvacrol, and vitamins (e.g., ascorbic and folic acid; Amin & Hosseinzadeh, 2016; Hannan et al., 2021; Jan et al., 2019). By adding different percentages of OL flour to cookies, Sahan et al. (2019) concluded that OL flour increased the phenolic compounds. According to Al‐Ansi et al. (2019), using BC in the biscuit caused an increase in the phenolic content of the product.

### 3.4 Antioxidant activity

**FIGURE 2:**
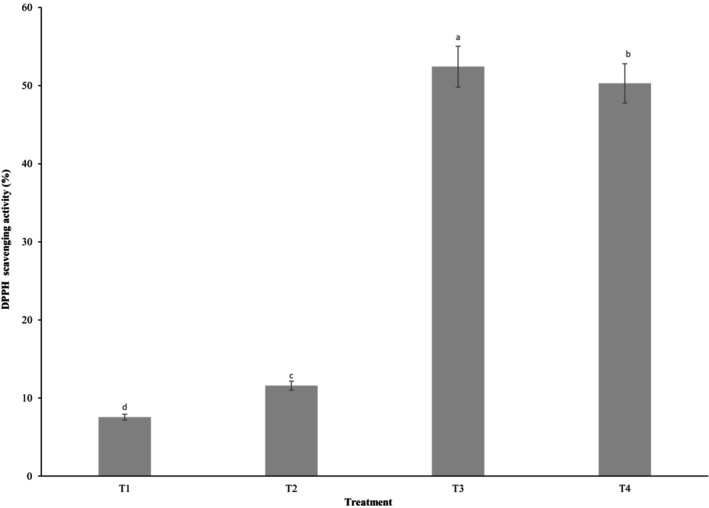
Antioxidant activity of breads containing a mixture of oleaster and BC flours. T_1_: Control, T_2_: 0.75% OL flour + 0.75% BC flour, T_3_: 1% OL flour + 1% BC flour, T_4_: 1.25% OL + 1.25% BC flour. Results are presented as a mean value ± SD, the different superscript letters are significant (*p* < .05).

The highest antioxidant activity (DPPH scavenging capacity) was observed in T3 sample (Figure 2). The increased antioxidant activity is due to the presence of flavonoid and phenolic compounds in BC and oleaster (Farzaei et al., 2015; Hannan et al., 2021). The lower antioxidant activity of wheat flour compared to oleaster flour, particularly BC flour (Table 2), caused an increase in the antioxidant capacity, in which BC flour played a more significant role. In the reports of Öztürk et al. (2018) and Sahan et al. (2019), OL flour increased the antioxidant activity in yogurt and cookies, respectively. The antioxidant activity of biscuits also increased with the addition of BC flour (Al‐Ansi et al., 2019).

### 3.5 Texture profile analysis

The results of the texture analysis are shown in Table 4. The hardness of the control (1.58 N) on the first day was not significantly different from the samples containing OL and BC flours (1.18–2.18 N; *p* > .05). The volume and porosity of bread are influencing parameters on the hardness of its texture. The bread with a smaller volume has a more compact texture and is harder (Mikulec et al., 2019). In this study, the hardness of each bread sample increased significantly during 6 days of storage due to staling (*p* < .05). On the sixth day, the bread samples containing 1.5% and 2.5% OL and BC flours had higher hardness than the control. However, no significant difference was observed between the control (2.96 N) and bread containing a 2% BC and OL mixture (3.45 N). Partial substitution of wheat with a gluten‐free flour especially in high amounts increases the hardness by diluting the gluten network. An increase in the texture hardness by substituting wheat with amaranth flour (50%; Miranda‐Ramos et al., 2019) and barley flour (higher than 30%; Sullivan et al., 2011) has been reported previously. Polar fat can also assist in the retaining of gas in the dough matrix and improve elasticity (Miranda‐Ramos et al., 2019). The hardness of T4 increased significantly after 6 days (5.11 N) compared to the samples with lower amounts of OL and BC flours. The reason can be attributed to the dominance of the effect of carbohydrates and starch in 1.25%:1.25% w/w of OL and BC mixture over the effect of fat and the starch retrogradation phenomenon.

**TABLE 4:** Changes in texture parameters of breads containing a mixture of oleaster and BC flours during storage.

Sample	Day	Hardness (N)	Springiness (mm)	Cohesiveness	Chewiness (mj)
T_1_	1	1.58 ± 0.38^de^	5.99 ± 0.45^abc^	0.57 ± 0.03^ab^	5.72 ± 1.27^de^
T_1_	4	1.75 ± 0.47^de^	5.55 ± 0.32^bc^	0.55 ± 0.03^ab^	4.70 ± 1.77^e^
T_1_	6	2.96 ± 0.6^bc^	5.78 ± 0.40^bc^	0.56 ± 0.05^ab^	9.54 ± 2.59^bc^
T_2_	1	1.98 ± 0.48^de^	5.38 ± 0/26^bc^	0.5 ± 0/04^bcd^	5.91 ± 1.71^cde^
T_2_	4	2.91 ± 0.56^bc^	5.88 ± 0.34^b^	0.53 ± 0.04^abc^	7.62 ± 1.04^bcde^
T_2_	6	4.88 ± 0.65^a^	5.90 ± 0.21^b^	0.5 ± 0.03^bcd^	10.02 ± 1.9^ab^
T_3_	1	1.18 ± 0.12^e^	5.85 ± 0.18^bc^	0.59 ± 0.03^a^	4.26 ± 1.1^e^
T_3_	4	2.24 ± 0.62^cde^	5.22 ± 0.7^c^	0.47 ± 0.01^cd^	5.38 ± 1.45^de^
T_3_	6	3.45 ± 0.27^b^	5.94 ± 0.3^abc^	0.43 ± 0.04^d^	8.85 ± 1.35^bcd^
T_4_	1	2.18 ± 0.15^cde^	6.63 ± 0.38^a^	0.6 ± 0.04^a^	8.57 ± 1.27^bcd^
T_4_	4	2.33 ± 0.47^cd^	5.91 ± 0.51^abc^	0.57 ± 0.09^ab^	8.7 ± 1.41^bcd^
T_4_	6	5.11 ± 0.71^a^	5.73 ± 0.24^bc^	0.48 ± 0.06^bcd^	12.97 ± 2.97^a^

*Note*: T_1_: Control, T_2_: 0.75% OL flour + 0.75% BC flour, T_3_: 1% OL flour + 1% BC flour, T_4_: 1.25% OL + 1.25% BC flour. Results are presented as a mean value ± SD, the different superscript letters are significant in the same column (*p* < .05).

The springiness of the samples was not significantly different on similar days of storage (*p* > .05) but decreased during the 6 days. The cohesiveness of the control and T2 did not change significantly with time, but there was a decreasing trend in T3 and T4 samples from the first to sixth days of storage. There was no significant difference between the chewiness of the control and the samples containing 2% of OL and BC flours on the same day of analysis (*p* > .05). The T4 on the sixth day of storage possessed the highest chewiness (12.97 mj), which was not significantly different from T3 (10.02 mj; *p* > .05; Table 4).

### 3.6 Color

The results of the effect of using OL and BC in bread formulation are shown in Table 5. The L* value of the bread samples decreased with an increase in the contribution of OL and BC mixture (from 52.3 in control to 42.88–43.24 in other samples), due to the darker color of BC and oleaster flour compared to wheat flour (Table 5). By adding OL and BC flours to the bread, the a* (redness) increased, and b* (yellowness) decreased. Similar results were reported by Sahan et al. (2019) and Al‐Ansi et al. (2019), with the addition of OL flour and BC flour in cookies and biscuits, respectively. The results of the present study indicated no significant difference in ∆E (*p* > .05). It is probably due to the low concentrations of OL and BC. If ∆E is less than 1, the color difference between the sample and the control cannot be seen by the eye. In values between 1 and 3, the existing color difference will not be easily seen, but if it is higher than 3, the color difference will be remarkable (Mikulec et al., 2019). The chroma value which indicates the color intensity was significantly lower in samples containing OL and BC flours (*p* < .05). It seems that it is related to a significant decrease in the yellowness compared to the control. Çakmakçı et al. (2015) also declared that the addition of OL flour to ice cream reduced the chroma.

**TABLE 5:** Color values of breads containing a mixture of oleaster and BC flours.

Sample	L*	a*	b*	∆*E*	Chroma
T_1_	52.3 ± 0.34^a^	−0.57 ± 0.04^b^	31.73 ± 0.25^a^	–	31.73 ± 0.2^a^
T_2_	42.88 ± 0.2^b^	2.63 ± 0.03^a^	26.81 ± 0.17^b^	11.25 ± 0.17^a^	26.94 ± 0.16^b^
T_3_	42.98 ± 0.4^b^	2.36 ± 0.38^a^	26.64 ± 0.16^b^	11.17 ± 0.39^a^	26.75 ± 0.17^b^
T_4_	43.24 ± 0.14^b^	2.40 ± 0.3^a^	26.7 ± 0.21^b^	10.93 ± 0.16^a^	26.81 ± 0.19^b^

*Note*: T_1_: Control, T_2_: 0.75% OL flour + 0.75% BC flour, T_3_: 1% OL flour + 1% BC flour, T_4_: 1.25% OL + 1.25% BC flour. Results are presented as a mean value ± SD, the different superscript letters are significant in the same column (*p* < .05).

### 3.7 Microstructure

**FIGURE 3:**
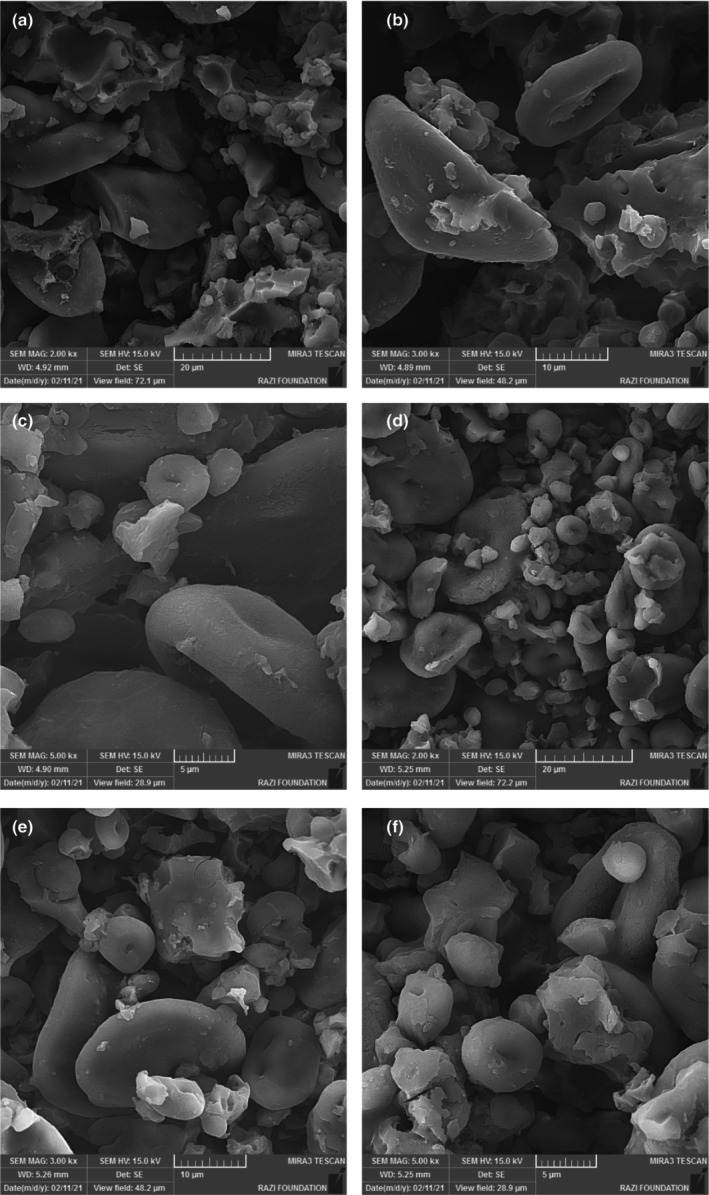
Scanning electron micrographs of bread samples. Control: A = 2.00 kx, B = 3.00 kx, and C = 5.00 kx; T3 1% OL flour + 1% BC flour: D = 2.00 kx, E = 3.00 kx, and F = 5.00 kx.

The microstructures of the control (A) and T3 sample (B) are shown in Figure 3. There were more empty spaces in the control bread. Empty spaces (black dots) represent bread cavities. In the microstructure of the B, a more compact texture was observed compared to the control. This is due to the aggregated mass of gelatinized starch particles and the filling of the remaining space with OL and BC flours. The presence of such a microstructure in the bread verified the textural characteristics compared to the control bread. Filling the empty spaces of the dough with the particles of OL and BC flours and the incapability of their protein to retain the gas produced by fermentation made the texture slightly more tough compared to the control.

### 3.8 Sensory evaluation

**FIGURE 4:**
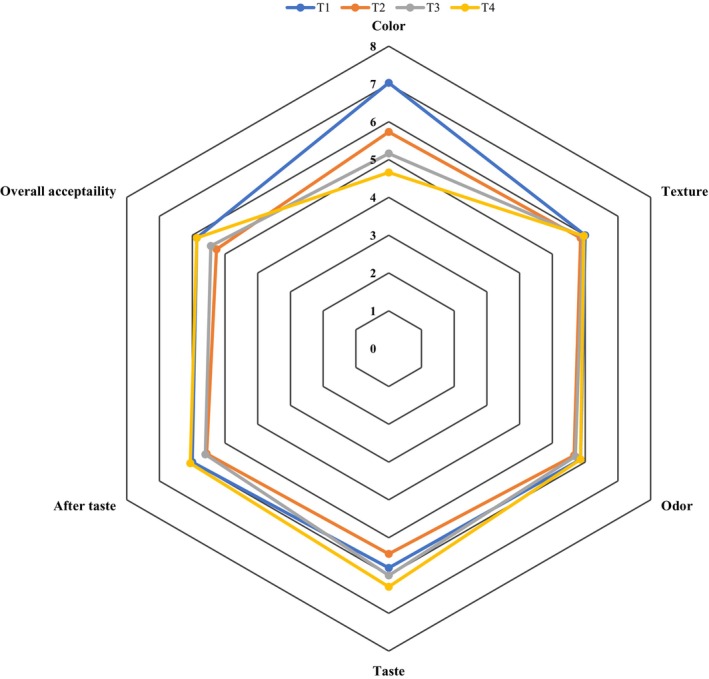
Sensorial attributes of breads containing a mixture of oleaster and BC flours. T_1_: Control, T_2_: 0.75% OL flour + 0.75% BC flour, T_3_: 1% OL flour + 1% BC flour, T_4_: 1.25% OL + 1.25% BC flour.

The sensory evaluation results in Figure 4 indicate no significant difference between the evaluated characteristics (*p* > .05), except for color. The color score decreased significantly by increasing the amount of OL and BC flours (*p* < .05). These results were in accordance with the sensory evaluation results of Nezamdoost‐sani et al. (2018) and Osman et al. (2015). Since OL has a specific astringency and BC may also have a specific aftertaste, we also evaluated the aftertaste in the sensory analysis. As shown in Figure 4, the aftertaste of none of the OL and BC‐containing samples was significantly different from the control. The reason seems to be the addition of relatively small amounts of OL and BC flour in the formulation of toast bread. Besides, it should be noted that the overall acceptability of OL‐ and BC‐containing bread samples was satisfying.

## 4 CONCLUSION

Based on the obtained results, developing a functional toast bread with suitable physicochemical characteristics is possible. Incorporating the combination of oleaster and BC flours improved the nutritional quality of bread. The amount of protein, calcium, and antioxidant properties increased significantly, particularly in T3 and T4 samples. The sensory evaluation results were acceptable, and the proper concentration for the simultaneous use of oleaster and BC flour was 2% in toast bread.

## AUTHOR CONTRIBUTIONS

**Saba Ghadarloo:** Investigation (equal); methodology (equal); writing – original draft (equal). **Samar Mansouripour:** Conceptualization (lead); data curation (lead); formal analysis (lead); project administration (lead); supervision (equal); validation (equal); writing – original draft (lead); writing – review and editing (lead). **Solmaz Saremnezhad:** Investigation (equal); methodology (equal); project administration (equal); supervision (equal); validation (equal); writing – review and editing (equal).

## FUNDING INFORMATION

This research received no specific grant from any funding source.

## CONFLICT OF INTEREST STATEMENT

The authors declare that there is no conflict of interest in this study.

## ETHICS STATEMENT

This study does not involve any human or animal testing.

## INFORMED CONSENT

Written informed consent was obtained from all study participants.

## Data Availability

The data that support the findings of this study are available from the corresponding author upon reasonable request.
